# Eosinophil Count Is a Common Factor for Complex Metabolic and Pulmonary Traits and Diseases: The LifeLines Cohort Study

**DOI:** 10.1371/journal.pone.0168480

**Published:** 2016-12-15

**Authors:** Marzyeh Amini, Dinara Bashirova, Bram P. Prins, Eva Corpeleijn, LifeLines Cohort Study, Marcel Bruinenberg, Lude Franke, Pim van der Harst, Gerjan Navis, Bruce H. R. Wolffenbuttel, Ronald P. Stolk, Cisca Wijmenga, Dirkje S. Postma, Gerard H. Koppelman, H. Marike Boezen, Judith Vonk, Harold Snieder, Behrooz Z. Alizadeh

**Affiliations:** 1 Department of Epidemiology, University Medical Center Groningen, University of Groningen, Groningen, The Netherlands; 2 LifeLines Cohort Study, University Medical Center Groningen, University of Groningen, Groningen, The Netherlands; 3 Department of Genetics, University Medical Center Groningen, University of Groningen, Groningen, The Netherlands; 4 Department of Cardiology, University Medical Center Groningen, University of Groningen, Groningen, The Netherlands; 5 Department of Nephrology, University Medical Center Groningen, University of Groningen, Groningen, The Netherlands; 6 Department of Endocrinology, University Medical Center Groningen, University of Groningen, Groningen, The Netherlands; 7 Department of Pulmonology, University Medical Center Groningen, University of Groningen, Groningen, The Netherlands; 8 Groningen Research Institute for Asthma and COPD, University Medical Center Groningen, University of Groningen, Groningen, The Netherlands; 9 Department of Pediatric Pulmonology and Pediatric Allergology, Beatrix Children’s Hospital, Groningen, The Netherlands; Tulane University School of Public Health and Tropical Medicine, UNITED STATES

## Abstract

There is ongoing debate on the association between eosinophil count and diseases, as previous studies were inconsistent. We studied the relationship of eosinophil count with 22 complex metabolic, cardiac, and pulmonary traits and diseases. From the population-based LifeLines Cohort Study (N = 167,729), 13,301 individuals were included. We focused on relationship of eosinophil count with three classes of metabolic (7 traits, 2 diseases), cardiac (6 traits, 2 diseases), and pulmonary (2 traits, 2 diseases) outcomes. Regression analyses were applied in overall, women and men, while adjusted for age, sex, BMI and smoking. A *p*-value of <0.00076 was considered statistically significant. 58.2% of population were women (mean±SD 51.3±11.1 years old). In overall, one-SD higher of ln-eosinophil count was associated with a 0.04 (±SE ±0.002;p = 6.0×10^−6^) SD higher levels in ln-BMI, 0.06 (±0.007;p = 3.1×10^−12^) SD in ln-TG, 0.04 (±0.003;p = 7.0×10^−6^) SD in TC, 0.04 (±0.004;p = 6.3×10^−7^) SD in LDL, 0.04 (±0.006;p = 6.0×10^−6^) SD in HbA1c; and with a 0.05 (±0.004;p = 1.7×10^−8^) SD lower levels in HDL, 0.05 (±0.007;p = 3.4×10^−23^) SD in FEV1, and 0.09 (±0.001;p = 6.6×10^−28^) SD in FEV1/FVC. A higher ln-eosinophil count was associated with 1.18 (95%CI 1.09–1.28;p = 2.0×10^−5^) odds ratio of obesity, 1.29 (1.19–1.39;p = 1.1×10^−10^) of metabolic syndrome, 1.40 (1.25–1.56;p = 2.7×10^−9^) of COPD and 1.81 (1.61–2.03;p = 1.0×10^−23^) of asthma. Similar results were found in women. We found no association between ln-eosinophil count either with blood pressure indices in overall, women and men; or with BMI, LDL, HbA1c and obesity in men. In a large population based cohort, we confirmed eosinophil count as a potential factor implicated in metabolic and pulmonary outcomes.

## Background

Eosinophils are specialized multifunctional leukocytes which constitute up to 4% of peripheral white blood cells [1–2]. They are involved in the regulation of immune response by acting as antigen-presenting cells and augmenting of the expression of pro-inflammatory or inhibitory cytokines [3–5]. As summarized in Table A in S1 File, a number of clinical and epidemiological studies have reported differential findings on the association of eosinophil count with complex traits and diseases in human. On the one hand, higher eosinophil count has been associated with increased coagulation factors level of serum fibrinogen and platelet count [4, 6], an increase in the risk and severity of coronary atherosclerosis [3, 4, 6], exacerbation of asthma [7–11], pulmonary hypertension [12], metabolic syndrome (MetS) [13], and inflammatory bowel diseases [14, 15]. On the other hand, a protective effect of higher eosinophil count has been reported on glucose homeostasis [16], risk of type 2 diabetes (T2D) [16–18], and hypertension (HTN) [18], as summarized in Table A in S1 File. Nevertheless, the eosinophil counts and its association with complex diseases are likely to be biased by disease modifying factors, such as age and gender [19–23]. Furthermore, the reproducibility of these associations is questionable as the corresponding studies, with a few exceptions, had included a relatively small number of subjects, and were performed across heterogeneous populations. This argument is endorsed as no assessment of the association between eosinophil count and different complex outcomes has been performed in the same homogenous population. Hence, the role of eosinophil count in complex diseases remains inconclusive (Table A in S1 File). Although the analyses of disease intermediate traits are instructive and powerful to unravel disease pathogenesis; little attention has been given to the relationship between eosinophil count and disease associated intermediate traits. One may therefore propose a population based cohort study offers an ideal setting to unearth the role of eosinophil count as an emerging universal risk factor for multifactorial disorders [24]. We aimed therefore to investigate whether eosinophil count is consistently associated with common complex intermediate traits and diseases in the baseline measurements of a randomly selected subpopulation of the LifeLines cohort study, in men as well as in women. LifeLines is a prospective population-based cohort study examining 167,729 residents of the Netherlands and aims to unravel how life-time exposure to environmental and genetic factors influences individual susceptibility to multifactorial diseases. In the present study, we examined the association of eosinophil count with 22 complex metabolic, cardiac, and pulmonary traits and disorders in 13,301 of total population (overall), in men and in women from the LifeLines study.

## Methods

### Population

The present study was conducted within the framework of the LifeLines Cohort Study. Details on study design and objectives of LifeLines have been described elsewhere [25, 26]. In brief, LifeLines is a prospective cohort study, covering three generations, including 167,729 residents of three Northern provinces of the Netherlands. It employs a broad range of procedures to assess the biomedical, socio-demographic, behavioural, physical and psychological factors, which contribute to health and disease of the general population, with a special focus on multi-morbidity and complex genetics. LifeLines is conducted according to the guidelines of the Declaration of Helsinki and all procedures involving human subjects were approved by the Medical Ethics Committee of the University Medical Center Groningen (UMCG). A written informed consent was obtained from all participants. For the present study, we included the third data release of LifeLines belonging to 13,301 unrelated individuals for whom dedicated detailed data on a wide variety of phenotypes were in the third release of LifeLines, and included blood measurements and genome-wide genotyping data.

### Outcomes

We focused on three classes of metabolic, cardiac, and pulmonary disorders and underlying intermediate traits.

The class of metabolic outcomes included the intermediate traits of body mass index (BMI, kg/m^2^), lipid profile (triglycerides [TG, mmol/L], total cholesterol [TC, mmol/L], high density lipoprotein [HDL, mmol/L], low density lipoprotein [LDL, mmol/L]), hemoglobin A1c (HbA1c, %), and fasting glucose (FG, mmol/L) as well as obesity, MetS and T2D. To calculate BMI, height and weight were measured without shoes and in light clothing to the nearest 0.1 cm and 0.1 kg, respectively, and BMI was calculated as weight/height squared (kg/m^2^) according to the National Heart, Lung and Blood Institute guidelines [27] and TG, TC, HDL and LDL were measured using enzymatic colorimetric assay (Modular Roche) [27]. FG was measured with a hexokinase method (Integra Roche) and the HbA1c level was measured using a turbidimetric inhibition immunoassay [27]. More details have been explained previously [28, 29]. Obesity was defined based on BMI≥30 (kg/m^2^) [30]. The presence of MetS was defined as the presence of three or more of the following four risk factors: a) abdominal obesity defined as waist circumference in men >102 cm and in women >88 cm; b) dyslipidemia defined as serum triglycerides≥1.7 mmol/L or pharmacologic treatment for elevated triglycerides and serum HDL cholesterol <1.03 mmol/L in men and <1.29 mmol/L in women or pharmacologic treatment for low HDL cholesterol; c) HTN defined as SBP≥130 mmHg or DBP≥85 mmHg or pharmacologic treatment for elevated blood pressure; d) hyperglycemia defined as FG≥5.6 mmol/L or pharmacologic treatment for elevated plasma glucose [31]. A diagnosis of T2D was assigned to any participant who had either self-reported T2D or use of anti-T2D medication(s) or a FG≥7.0 mmol/L or an HbA1c≥6.5% [32–35]. This algorithm is explained in detail in the Methods I in S1 File and Figs A and B in S1 File.

The exclusion criterion for the analyses of TG, TC, HDL, and LDL was the use of lipid lowering medication. For the analyses on fasting glucose, exclusion criteria were doctor's diagnosed T2D, use of anti-T2D medications and people who a FG≥7 mmol/L or an HbA1c≥6.5%.

The class of cardiac traits and diseases included systolic blood pressure (SBP, mmHg), diastolic blood pressure (DBP, mmHg), mean arterial pressure (MAP, mmHg), pulse pressure (PP, mmHg), estimating glomerular filtration rate (eGFR, mL/min/1.73 m2), urine albumin-to-creatinine ratio (UACR, mg/mmol) as well as HTN and myocardial infarction (MI). SBP and DBP of participants were recorded by an automatic blood pressure monitor (DinaMap, PRO 100V2) every minute during 10 minutes. This resulted in 10 blood pressure measures per participant. The mean of the last three measures was used for calculating blood pressure per participant. For all individuals taking antihypertensive or blood pressure lowering medication, imputed SBP and DBP were calculated based on the guidelines of the international consortium on blood pressure (ICBP) [36] by adding 15mmHg to the measured SBP level and 10mmHg to the measured DBP level. Using the imputed SBP and DBP values, MAP and PP were calculated based on following formulas: MAP = (2DBP+SBP)/3 and PP = SBP-DBP [36]. CKD-EPI equation was used for estimating GFR which expressed for specified race, sex and serum creatinine in mg/dl based on the study by Levey *et al*. [37, 38]. UACR is a ratio between the urine albumin (BCG, colorimetric assay; Modular Roche) and the urine creatinine (enzymatic, IDMS traceable; Modular Roche) [38]. HTN was defined as a SBP≥140 mmHg and/or a DBP≥90 mmHg or used anti-hypertensive medication [39]. MI was defined as a positive answer to the question “Have you ever had a myocardial infarction?”. Exclusion criteria for the analyses of blood pressure indices were the presence of self-reported MI or doctor's diagnosed heart failure or coronary artery disease.

The class of pulmonary traits and diseases included forced expiratory volume in one second (FEV_1_, L), ratio of FEV_1_ and forced vital capacity (FEV_1_/FVC) as well as chronic obstructive pulmonary disease (COPD) and asthma. FEV_1_ and FVC were measured by a spirometer (Welch Allyn version 1.6.0.489, PC-Based SpiroPerfect with CardioPerfect Workstation software) following the American Thoracic Society criteria [40]. According to the test criteria the difference between the best and the next best FEV_1_ and FVC should not exceed 150 ml. If the difference was greater than 150 ml, the test was repeated. The presence of COPD was defined as a FEV_1_/FVC ratio<70% in ever (ex- or current) smokers with age≥40 years old; Asthma was defined as a clinical diagnosis of asthma, or at least two asthma symptoms (wheeze, attack at rest, woken by an attack) in addition to asthma medication use (Methods II in S1 File and Fig C in S1 File) [41, 42].

### Determinants

#### Eosinophil

Eosinophil count (×10^3^ cells/μl) were measured using Automated Hematology Blood Analyzer (XE2100–system; Sysmex, Japan). In the main analyses, absolute eosinophil count was utilized. Absolute eosinophil count was calculated by multiplying the relative eosinophil count by the total leukocyte count.

#### Modifiers

Age and sex were recorded according to the community registry database. Smoking status was reported as nonsmoker, former smoker, or current smoker. The number of pack-years was calculated based on self-reported information on the number of cigarettes smoked per day and the number of years smoked. Pack-years is the number of years with 20 cigarettes per day.

### Data analysis

Eosinophil count, BMI, TG, SBP, DBP, MAP, and PP were not normally distributed. To test for normality distribution, skewness test were used. If skewness was between -0.5 and 0.5, the distribution is approximately normally distributed; otherwise, a natural logarithmic transformation (ln) was carried out to achieve approximate normality and fulfill regression assumptions for these variables. These variables were presented as median with inter-quartile range in the descriptive table. TC, HDL, LDL, HbA1c, FG, FEV_1_, and FEV_1_/FVC ratio were normally distributed and were presented as mean±standard deviation (SD). Because of previous reports on gender differences in eosinophil count and complex diseases, all analysis models were stratified by gender. Variables were compared between men and women using independent *t* tests or Mann-Whitney U tests when appropriate. Categorical variables were shown as frequency with percentage and were compared by χ^2^ test.

Multivariate linear regression analyses were performed to evaluate the relationship between blood eosinophil count and each of the studied intermediate traits. The analyses were adjusted for the potential confounding effect of major baseline characteristics including age and sex. To correct for a potential non-linear relationship of age with outcomes, we added also square of age in models. Next for each classes, additional covariables were added when appropriate, including BMI and smoking habit in metabolic and cardiac classes. Additionally, SBP, DBP, height, overweight and obesity were adjusted in eGFR and UACR models. Besides, smoking habit and height were added in pulmonary class. The association of eosinophil count and intermediate traits was reported by standardized coefficient (Sβ) which means a one-standard deviation higher eosinophil count would result in a β × SD change in the outcome variables.

Multivariate logistic regression analyses were performed to obtain the association of ln-transformed eosinophil count as independent variable and diseases outcomes. Beside age, age^2^ and sex as potential confounders, we added BMI as a confounder in T2D, HTN and MI diseases as well as smoking habit in all diseases outcomes and height in COPD and asthma diseases. The relation of eosinophil count and diseases outcomes was reported as odds ratio (OR) with the corresponding 95% confidence intervals (95% CIs). Since the study was nested within a population-based cohort study, OR was considered as approximation for fold increase risk of studies outcomes [43].

The Bonferroni correction for multiple testing was employed yielding a significance threshold of p-value<0.00076 (conservatively calculated as 0.05/66, which 66 being the total number of tests for 22 outcomes tested in three groups [overall, men and women]). Data analyses were performed by using SPSS statistical software (version 22; SPSS Inc., Chicago, USA).

## Results

The baseline characteristics of the total population and stratified by gender are presented in Table 1. Out of 13,301 (mean±SD age 51.3±11.1 years old) participants, 58.2% were women (51.1±10.9 years old) and men were slightly older (51.6±11.3 years old). Overall, 6.4% and 22.8% of participants reported using lipid lowering and antihypertensive medication, respectively, and 22.3% were current smokers with a mean of 13.3 (±SD 11.7) pack-years. The median eosinophil count was 0.16 (×10^3^ cells/μl) in overall and was significantly (p<0.001) higher in men (0.17; range 0.11–0.25) compared to women (0.15; 0.10–0.22). The level of the majority of the intermediate traits (with the exception of HDL and FEV_1_/FVC) was significantly higher in men than women (Table 1). Overall, the prevalence of HTN was 29.5%, followed by MetS (17.6%), COPD (9.5%), asthma (7.3%), T2D (3.8%) and MI (1.4%). The prevalence of the major disease outcomes (Table 1) in men was higher than women for MetS (19.4% vs. 16.2%;p<0.001), T2D (4.6% vs. 3.3%;p<0.001), HTN (36.3% vs. 24.6%;p<0.001), MI (2.6% vs. 0.6%;p<0.001), and COPD (11.0% vs. 8.4%;p<0.001). Obesity and asthma were more prevalent in women than men (17.2% vs. 15.3% and 7.6% vs. 6.8%; respectively; Table 1).

**Table 1:** Characteristics of the study population (N = 13,301).

Mean ± SD/ Median (interquartile range)/ n (%)			
	Overall (n = 13,301)	Men (n = 5,557)	Women (n = 7,744) ^#^
Gender: n (Female %)	7,744 (58.22)	-	-
Age (yrs.)	51.32 ± 11.10	51.65 ± 11.34	51.09 ± 10.92*
Lipid lowering medication: n (%)	851 (6.40)	448 (8.06)	403 (5.20)***
Antihypertensive medication: n (%)	3,029 (22.78)	1,294 (23.28)	1,735 (22.40)
Current smoking: n (%)	2,973 (22.35)	1,247 (22.44)	1,726 (22.28)
Pack-years	13.29 ± 11.67	13.64 ± 11.90	13.03 ± 11.48*
Eosinophils (×10^3^cells/μl)	0.16 (0.10–0.23)	0.17 (0.11–0.25)	0.15 (0.10–0.22)***
BMI (kg/m^2^)	25.77 (13.88–28.60)	26.25 (24.28–28.54)	25.30 (22.86–28.40)***
Normal (<25, %)	5,500 (41.37)	1,863 (33.53)	3,637 (47.00)***
Overweight (25≤BMI<30, %)	5,610 (42.20)	2,842 (51.15)	2,768 (35.77)
Obese (≥30, %)	2,185 (16.43)	851 (15.32)	1,334 (17.24)
Triglycerides (mmol/L)	1.04 (0.05–1.47)	1.23 (0.89–1.78)	0.94 (0.71–1.32)***
Total Cholesterol (mmol/L)	5.13 ± 0.99	5.18 ± 0.98	5.07 ± 1.00***
High-density lipoprotein (mmol/L)	1.45 ± 0.39	1.27 ± 0.31	1.57 ± 0.38***
Low-density lipoprotein (mmol/L)	3.32 ± 0.89	3.42 ± 0.87	3.18 ± 0.90***
HbA1c (%)	5.51 ± 0.34	5.52± 0.33	5.50 ± 0.34***
Fasting glucose (mmol/L)	4.99 ± 0.56	5.12 ± 0.56	4.89 ± 0.54***
Imputed SBP (mmHg) ^†^	130.00 (86.00–237.00)	135.00 (125.00–146.00)	125.00 (115.00–139.00)***
Imputed DBP (mmHg) ^†^	76.00 (35.00–150.00)	79.00 (73.00–87.00)	74.00 (68.00–82.00)***
Mean Arterial Pressure (mmHg)	94.33 (59.00–178.00)	97.67 (91.00–106.33)	91.00 (84.33–100.66)***
Pulse Pressure (mmHg)	53.00 (22.00–124.00)	55.00 (48.00–63.00)	51.00 (43.00–60.00)***
eGFR (mL/min/1.73 m^2^)	93.43 ± 14.75	94.49 ± 14.83	92.67 ± 14.65***
UACR (mg/mmol)	0.20 (0.11–0.34)	0.18 (0.10–0.31)	0.22 (0.13–0.37)***
FEV_1_ (L)	3.38 ± 0.83	4.01 ± 0.76	2.94 ± 0.55***
FEV_1_/FVC	0.76 ± 0.07	0.75 ± 0.08	0.77 ± 0.07***
Metabolic syndrome: n (%)^a^	2,337 (17.57)	1,079 (19.41)	1,258 (16.24)***
T2D: n (%)^b^	507 (3.81)	257 (4.62)	250 (3.28)***
Hypertension: n (%)^c^	3,921 (29.47)	2,019 (36.33)	1,902 (24.56)***
Myocardial infarction: n (%)^d^	190 (1.43)	143 (2.57)	47 (0.61)***
COPD: n (%)^e^	1,265 (9.51)	611 (11.00)	654 (8.44)***
Asthma: n (%)^f^	967 (7.27)	379 (6.82)	588 (7.60)***

**Abbreviations: BMI**: body mass index, **HbA1c:** Hemoglobin A1c, **SBP**: systolic blood pressure, **DBP**: diastolic blood pressure, **eGFR:** estimated glomerular filtration rate, **UACR:** Urine Albumin-to-Creatinine Ratio, **FEV**_**1**_: Forced expiratory volume in one second, **FVC**: Forced vital capacity, **COPD**: Chronic Obstructive Pulmonary Disease.

^**†**^ **Imputed SBP and DBP** were calculated as a following: For all individuals who taking antihypertensive or blood pressure lowering medication, were added 15mmHg to the measured SBP level, and 10mmHg to the measured DBP level. For individuals not taking such medication, the imputed values were left equal to the measured level.

^**a**^ **Metabolic syndrome** was defined as the presence of three or more of the following four traits: 1) abdominal obesity defined as waist circumference in men >102 cm and in women >88 cm; 2) dyslipidemia determined as serum triglycerides≥1.7 mmol/L or pharmacologic treatment for elevated triglycerides and serum HDL cholesterol <1.03 mmol/L in men and <1.29 mmol/L in women or pharmacologic treatment for low HDL cholesterol; 3) hypertension defined as either SBP≥130 mmHg or DBP≥85 mmHg or pharmacologic treatment for elevated blood pressure; 4) hyperglycemia determined as fasting glucose≥5.6 mmol/L or pharmacologic treatment for elevated plasma glucose.

^**b**^ **Type 2 diabetes** was defined by either clinical diagnosis, self–reported type 2 diabetes, type 2 diabetes pharmacologic treatment or undiagnosed type 2 diabetes defined by FG≥7.0 mmol/L or HbA1c≥6.5%).

^**c**^ **Hypertension** was defined as SBP≥140 mmHg and/or DBP≥90 mmHg or anti-hypertension medication use.

^**d**^ **Myocardial infarction** was based on self-reported.

^**e**^ **COPD** was based on FEV_1_/FVC ratio <70% and being an ever smoker (ex- or current smoker).

^**f**^ **Asthma** was based on a clinical diagnosis of asthma or two or more of the symptoms wheeze, attack at rest, woken by an attack and asthma medication use.

^**#**^ Women compared to men:

* *p*<0.05,

***p<0.001.

Table 2 shows the multivariate regression results of eosinophil count with metabolic traits and diseases in overall, in men and in women. A one-SD higher ln-transformed eosinophil count was significantly associated with 0.04 (±SE 0.002;p = 6.0×10^−6^) SD higher ln-transformed BMI, and also with a higher levels of ln-transformed TG, TC, LDL, and HbA1c. In contrast, it was significantly associated with 0.05 (±SE 0.004;p = 1.7×10^−8^) SD lower levels of HDL. When we adjusted our full model for total leukocytes count, only TC (Sβ±SE 0.04±0.003;p = 1.7×10^−4^) and LDL (Sβ±SE 0.04±0.004;p = 3.3×10^−5^) remained significant (Table B in S1 File, Model III). Stratified analysis showed that ln-transformed eosinophil count was significantly associated with higher levels of ln-transformed TG, TC, LDL and with lower levels of HDL in both men and women; while it was significantly associated with higher ln-transformed BMI levels only in men, and with higher HbA1c only in women (Table 2). Logistic regression analyses showed that a one-unit higher ln-transformed eosinophil count was significantly associated with an (OR) 1.18 (95% CI 1.09–1.28;p = 2.0×10^−5^) fold increase risk of obesity and 1.29 (1.19–1.39;p = 1.1×10^−10^) fold increase risk of MetS. In men, a one-unit higher ln-transformed eosinophil count was significantly associated with the increased risk of MetS (OR 1.29, 95% CI 1.19–1.39;p = 1.1×10^−10^); while in women, with the increased risk of obesity (OR 1.22, 95% CI 1.10–1.35;p = 1.3×10^−4^) and MetS (OR 1.34, 95% CI 1.20–1.50;p = 1.0×10^−7^).

**Table 2:** Multivariate regression results of ln-transformed eosinophil count with studied intermediate traits and diseases in metabolic class.

	Overall^#1^			Men			Women		
	**Sβ ± SE**^#2^	***p-*value**	**N**	**Sβ ± SE**^#2^	***p-*value**	**N**	**Sβ ± SE**^#2^	***p*-value**	**N**
ln-transformed BMI	0.04 ± 0.002	6.0 × 10^−6^	11,789	0.01 ± 0.003	0.308	4,908	0.06 ± 0.003	1.0 × 10^−6^	6,881
ln-transformed Triglycerides	0.06 ± 0.007	3.1 × 10^−12^	10,661	0.06 ± 0.012	5.0 × 10^−6^	4,376	0.07 ± 0.009	1.6 × 10^−8^	6,285
Total Cholesterol	0.04 ± 0.003	7.0 × 10^−6^	10,661	0.05 ± 0.005	3.8 × 10^−4^	4,376	0.03 ± 0.004	3.0 × 10^−3^	6,285
High-Density Lipoprotein	-0.05 ± 0.004	1.7 × 10^−8^	10,660	-0.05 ± 0.006	4.0 × 10^−4^	4,375	-0.06 ± 0.005	2.0 × 10^−6^	6,285
Low-Density Lipoprotein	0.04 ± 0.004	6.3 × 10^−7^	10,661	0.04 ± 0.006	4.0×10^−3^	4,376	0.05 ± 0.002	2.9 × 10^−5^	6,285
HbA1c	0.04 ± 0.006	6.0 × 10^−6^	11,751	0.03 ± 0.011	0.010	4,892	0.04 ± 0.008	1.1 × 10^−4^	6,859
Fasting glucose	0.008 ± 0.013	0.401	10,317	-0.008 ± 0.022	0.578	4,316	0.02 ± 0.015	0.045	6,001
	**OR (95% CI)**	***p-*value**	**Case/Non Case**	**OR (95% CI)**	***p-*value**	**Case/Non Case**	**OR (95% CI)**	***p-*value**	**Case/Non Case**
Obesity	1.18 (1.09–1.28)	2.0 × 10^−5^	2,058/10,480	1.14 (1.00–1.29)	0.040	804/4,444	1.22 (1.10–1.35)	1.3 × 10^−4^	1,254/6,036
Metabolic Syndrome	1.29 (1.19–1.39)	1.1 × 10^−10^	2,198/10,317	1.21 (1.08–1.35)	1.0 × 10^−3^	1,016/4,233	1.34 (1.20–1.50)	1.0 × 10^−7^	1,182/6,084
Type 2 diabetes	1.05 (0.91–1.20)	0.496	470/10,880	1.03 (0.85–1.25)	0.750	243/4,623	1.07 (0.88–1.31)	0.470	227/6,257

**Abbreviations: Sβ:** standardized coefficient, **BMI**: body mass index, **HbA1c:** hemoglobin A1c.

^**#1**^: All analysis models on intermediate traits were adjusted for confounding effect of age, age^2^, sex, BMI (with exception on BMI) and smoking habit. Analysis models on diseases were adjusted for confounding effect of age, age^2^, sex, and smoking habit; as well as, BMI also was adjusted on type 2 diabetes. Sex effect only included in overall model.

^**#2**^: Standardized coefficient (Sβ) means a one-standard deviation higher eosinophil count would result in a β x SD change in the outcome variables.

No significant association was found between ln-transformed eosinophil count and intermediate traits or diseases in the cardiac class including blood pressure and renal function indices as well as MI and HTN either in overall or when the analyses were stratified by gender (Table 3 and Table C in S1 File).

**Table 3:** Multivariate regression results of ln-transformed eosinophil count with studied intermediate traits and diseases in cardiac class.

	Overall^#1^			Men			Women		
	**Sβ ± SE**^#2^	***p*-value**	**N**	**Sβ ± SE**^#2^	***p-* value**	**N**	**Sβ ± SE**^#2^	***p-*value**	**N**
ln-transformed SBP	0.007 ± 0.002	0.345	11,625	0.007 ± 0.002	0.570	4,787	0.008 ± 0.002	0.419	6,838
ln-transformed DBP	0.01 ± 0.002	0.244	11,625	0.008 ± 0.003	0.381	4,787	0.01 ± 0.002	0.197	6,838
ln-transformed MAP	0.009 ± 0.002	0.250	11,625	0.008 ± 0.002	0.534	4,787	0.01 ± 0.002	0.246	6,838
Pulse Pressure	0.003 ± 0.168	0.749	11,786	0.006 ± 0.247	0.627	4,907	0.0004 ± 0.224	0.596	6,879
eGFR	-0.003 ± 0.167	0.616	11,560	0.004 ± 0.242	0.685	4,905	0.009 ± 0.227	0.332	6,655
ln-transformed UACR	-0.011 ± 0.016	0.025	11,561	-0.003 ± 0.025	0.798	4,903	-0.015 ± 0.020	0.182	6,658
	**OR (95% CI)**	***p-*value**	**Case/Non Case**	**OR (95% CI)**	***p-*value**	**Case/Non Case**	**OR (95% CI)**	***p-*value**	**Case/Non Case**
Hypertension	1.02 (0.95–1.10)	0.517	3,029/8,437	1.02 (0.92–1.12)	0.771	1,851/3,268	1.04 (0.94–1.15)	0.408	1,178/5,169
Myocardial Infarction	1.46 (1.12–1.90)	0.041	170/12,267	1.46 (1.07–1.99)	0.016	127/5,074	1.46 (0.87–2.45)	0.144	43/7,193

**Abbreviations: Sβ:** standardized coefficient, **SBP:** systolic blood pressure, **DBP:** diastolic blood pressure, **MAP:** mean arterial pressure, **eGFR:** estimated glomerular filtration rate, **UACR:** Urine Albumin-to-Creatinine Ratio.

^**#1**^: All analysis models on intermediate traits and diseases were adjusted for confounding effect of age, age^2^, sex, BMI and smoking habit. Additionally, SBP, DBP, height, overweight and obesity were adjusted in eGFR and UACR models. Sex effect only included in overall model.

^**#2**^: Standardized coefficient (Sβ) means a one-standard deviation higher eosinophil count would result in a β × SD change in the outcome variables.

As Table 4 presents, we found a one-SD higher of ln-transformed eosinophil count was highly significantly associated with a lower FEV_1_ and FEV_1_/FVC ratio. Logistic regression analyses showed that a higher ln-transformed eosinophil count was significantly associated with the increased risk of COPD (OR 1.40, 95% CI 1.25–1.56;p = 2.7×10^−9^) and asthma (OR 1.81, 95% CI 1.61–2.03;p = 1.0×10^−23^). By inclusion of total leukocyte count, the observed significant associations between eosinophil count and pulmonary traits and diseases remained still significant at a p<7.6×10^−4^ (Table D in S1 File, Model III). These results remained significant in men as well as in women (Table 4). In addition evaluation of the relationship between eosinophil count and the intermediate outcomes using partial correlation analysis while controlling for covariates effect, shows the similar results to the multivariate regression analyses (Table E in S1 File).

**Table 4:** Multivariate regression result of ln-transformed eosinophil count with studied intermediate traits and diseases in pulmonary class.

	Overall^#1^			Men			Women		
	**Sβ ± SE**^#2^	***p-*value**	**N**	**Sβ ± SE**^#2^	***p-*value**	**N**	**Sβ ± SE**^#2^	***p-*value**	**N**
FEV_1_	-0.05 ± 0.007	3.4 × 10^−23^	11,849	-0.06 ± 0.013	7.5 × 10^−9^	4,903	-0.07 ± 0.008	2.3 × 10^−17^	6,946
FEV_1_/FVC	-0.09 ± 0.001	6.6 × 10^−28^	11,851	-0.09 ± 0.002	2.1 × 10^−12^	4,905	-0.09 ± 0.001	1.9 × 10^−16^	6,946
	**OR (95% CI)**	***p-*value**	**Case/Non Case**	**OR (95% CI)**	***p-*value**	**Case/Non Case**	**OR (95% CI)**	***p-*value**	**Case/Non Case**
COPD	1.40 (1.25–1.56)	2.7 × 10^−9^	1,066/10,342	1.40 (1.19–1.64)	3.6 × 10^−5^	521/4,200	1.40 (1.20–1.62)	2.3 × 10^−5^	545/6,142
Asthma	1.81 (1.61–2.03)	1.0 × 10^−23^	869/10,922	1.70 (1.40–2.04)	2.3 × 10^−8^	334/4,575	1.89 (1.63–2.20)	4.6 × 10^−17^	535/6,347

**Abbreviations: Sβ:** standardized coefficient, **FEV**_**1**_: Forced expiratory volume in one second, **FVC:** Forced vital capacity.

^**#1**^: All analysis models on intermediate traits and diseases were adjusted for confounding effect of age, age^2^, sex, smoking habit, and height. Sex effect only included in overall model.

^**#2**^: Standardized coefficient (Sβ) means a one-standard deviation higher eosinophil count would result in a β × SD change in the outcome variables.

## Discussion

In the large population based LifeLines Cohort Study, we investigated the association of eosinophil count with complex diseases and their underlying intermediate phenotypes. We found that higher levels of BMI, TG, TC, LDL, and HbA1c and lower levels of HDL, FEV1, and FEV1/FVC were significantly associated with higher eosinophil count. We found no association between eosinophil count and FG, blood pressure and renal function indices. Further we observed that higher eosinophil count was significantly associated with a higher prevalence of obesity, MetS, COPD, and asthma, but not with T2D, HTN and MI. In stratified by gender analyses, similar results to overall population were found in women. While, in men higher eosinophil count was significantly associated with higher levels of BMI, TG, TC, and lower levels of HDL; in addition to higher prevalence of MetS, COPD, and asthma. Taking together, in comparison to previous studies presented in Table A in S1 File, using basic models and full models we could find consistently significant association of eosinophil count with traits and diseases in metabolic and pulmonary classes.

In the present study, eosinophil count was significantly higher in participants with higher BMI, triglyceride, cholesterol, LDL, and lower HDL level. Besides, higher eosinophil count was significantly associated with higher BMI in women. Lately, several studies have reported inconsistent associations between eosinophil count and metabolic traits, as was summarized in Table A in S1 File. Our findings are in line with one study which reported that eosinophil count was significantly higher in people with hyper-triglyceridemia and hyper-cholesterolemia [44] and a previously reported significant reverse association between eosinophil count and HDL level [45]. Another study found no association between eosinophil count and high-LDL level [46]. Consistent with a previous reported association between eosinophil count and lipid indices in our data and those from others [45, 46], participants with higher eosinophil count showed a significantly higher risk for obesity and MetS. These findings were in concordance with those of previous studies as summarized in Table A in S1 File [13, 18, 47–49] on MetS [13, 18, 45, 49] in Asian (Korean) [13, 45] and in Caucasian (Spanish) populations [49]. Taken together, our findings corroborate those of previous reports and confirm that a higher eosinophil count is significantly associated with a higher level of lipid indices such as high TC, high LDL, high TG, and low HDL level and with higher risk for obesity and MetS. Higher levels of eosinophil counts seems to be a significant risk factor for metabolic disorders associated with lipid disturbances.

We found no significant association between eosinophil count and FG; likewise, eosinophil count showed no significant relationship to T2D. To date, few studies have described the relationship between eosinophil count and glucose level. The case- control study by Xu *et al*. and the cross-sectional study by Zhu *et al*. found that a lower eosinophil count was associated with hyperglycemia [17, 50]. As summarized in Table A in S1 File, others also found no significant relationship between eosinophil count and the risk for T2D [18, 47, 48]. However, one cross-sectional study found that the mean eosinophil count increased with progression of diabetes [51], which is likely to be a reflection of disease severity. Taken together, our finding of a lack of association between eosinophil count and glycaemia adhere with earlier reports of no significant association between eosinophil count and T2D. Therefore, it is unlikely that eosinophil count is a strong determinant of glycaemia or T2D and to play a major role in glycemic control.

We found no association between eosinophil count and SBP, DBP, PP, MAP, HTN or MI. Likewise, a few previous epidemiological studies (Table A in S1 File) were inconsistent on association between eosinophil count and HTN. While one study found no significant association between eosinophil count and HTN [18]; another study has reported otherwise [13]. Unlike our finding, a few reports showed that higher eosinophil count was associated with coronary heart disease [6, 52, 53]. In our study the association of eosinophil count and MI did not reach significance after multiple testing correction. Observing this conflicting result with our findings, we investigated the power of our study. Indeed we estimated that this study had a suboptimal power of 54.1% to detect the association of eosinophil count with MI due to a low number of subjects with MI (N = 190, Table 1) in our study. When we additionally explored the association of indices of renal function, which are closely related to cardiovascular parameters, we observed no relationship between eosinophil count and eGFR and UACR as well. The lack of association of renal parameters and hypertension, as opposed to the association with metabolic traits, remains interesting in terms of pathway dissection—as clinically the metabolic syndrome, hypertension and renal damage are very closely associated and this might be at the phenotypic level. Putting together, though eosinophil count is unlikely to be significantly associated with the indices of blood pressure, the hypothesis that higher eosinophil count is a risk factor for MI remains yet to be addressed. Future well-powered population based longitudinal studies are required to test this hypothesis.

Our results showed that a higher eosinophil count was significantly associated with a lower FEV_1_ and FEV_1_/FVC ratio in multivariate models controlling for potential confounders. We have also previously reported that asthmatic patients with lower FEV_1_ had higher eosinophil count in blood [23, 54] and in sputum [55, 56], which endorsed previous studies on association of eosinophil count with low lung function in asthmatic patients [57–60]. Also, we have previously shown that the pattern and strength of the association between eosinophil and lung function differs according to gender, smoking habit, and presence of skin test reactivity [23, 54]. Additionally, we found that a higher eosinophil count was linked to higher risk for asthma and COPD. Our findings were consistent with previous studies which showed, eosinophil level were higher in asthmatic [60–62] and COPD [62–64] patients. Nevertheless, treatment strategy directed at normalization of eosinophil remains inconclusive regarding the critical role of eosinophil in asthma and COPD pathogenesis [65–67]. Taken together, our findings align with those aforementioned studies (Table A in S1 File) suggesting that eosinophil count is likely to be a risk factor for low lung function, airway obstruction, COPD and asthma. Blood eosinophils are derived from bone marrow myeloid progenitors, and they maturate in the peripheral circulation. The main factor regulating eosinophils is interleukin-5, but also other cytokines are important in eosinophil survival, such as IL3 and GMCSF [68]. Eosinophils traffic to the site of inflammation and can have important local effects, e.g. asthma in the airway.

The relation between the chronic disease and blood eosinophils can be twofold, either eosinophils being part of the causal pathway, or diseases that are associated with local production of cytokines which then stimulate increase of eosinophil count in the peripheral circulation (i.e. chemokines produced by adipose tissue)[69]. In the present study, we focused on eosinophil count and found strong associations with metabolic and pulmonary outcomes which fall within aforementioned previous observations (Table A in S1 File). When studying the role of inflammatory multifunctional cells in complex diseases, the complex interaction out of functional dependencies among these cells is a limiting factor for definite conclusions. Nevertheless, the findings remained significant for associations between eosinophil count and TC, LDL, pulmonary outcomes, even when we corrected for leukocytes count. This suggests despite a labyrinthine functional dependency between various type of leukocytes, eosinophil count maintained its independent effects on these outcomes. For other metabolic traits and diseases the significant findings presented in the Table 2, turned to be statistically non-significant after including leukocyte count into the regression model. We therefore propose proper follow-up functional or methodological oriented studies such as mediation and moderation analyses using longitudinal observational studies, and a causal modeling such as Mendelian randomization approach to distinguish between causal, pleiotropic or co-stimulatory nature of observed significant associations between eosinophil count and the studied complex outcomes.

### Limitations

This study has a number of limitations; firstly, it remains to be tested whether the association of eosinophil count with traits and diseases was independent of other inflammatory biomarkers; a next is whether the identified significant associations between eosinophil count and traits and disease outcomes are causal. Appropriate approaches, such as the use of genetic determinants of eosinophil count, are required to test the causal nature of these associations. Thirdly, our study is cross sectional; longitudinal measures of eosinophil count with the setting of prospective cohort studies are required to further validate and specify the role of eosinophils in the development of complex diseases.

## Conclusion

Higher eosinophil count is significantly associated with metabolic traits including BMI, TG, TC, HDL, LDL, HbA1c, and with lung function indices such as FEV_1_, and FEV_1_/FVC level, and is likely to be a common factor for obesity, MetS, COPD, and asthma and perhaps for MI but not for HTN. These association hold in both men and women, with an exception of obesity. Hence, higher eosinophil count may serve as an informative biomarker to identify individuals at higher risk for associated complex diseases and may have an additive predictive value when combined with known predictors. Future large scale genetic and cellular studies preferably embedded within longitudinal cohorts are needed to establish the causal nature of eosinophil count in complex traits and diseases. When confirmed, these findings may ultimately have a clinical implication such as prediction or intervention, which remains to be determined.

## Supporting Information

S1 File Supplementary Text and Figures. (DOCX)

## Abbreviations

BMI: body mass index
CIs: confidence intervals
COPD: chronic obstructive pulmonary disease
DBP: diastolic blood pressure
eGFR: Estimated Glomerular Filtration Rate
FEV1: forced expiratory volume in one second
FG: fasting glucose
FVC: forced vital capacity
HbA1c: hemoglobin A1c
HDL: high density lipoprotein
HTN: hypertension
ICBP: international consortium on blood pressure
LDL: low density lipoprotein
ln: natural logarithmic
MAP: mean arterial pressure
MetS: metabolic syndrome
MI: myocardial infarction
OR: odds ratio
PP: pulse pressure
Sβ: standardized coefficient
SBP: systolic blood pressure
SD: standard deviation
T2D: type 2 diabetes
TC: total cholesterol
TG: triglycerides
UACR: Urine Albumin-to-Creatinine Ratio
