# Supplementary material for: Eosinophil Count Is a Common Factor for Complex Metabolic and Pulmonary Traits and Diseases: The LifeLines Cohort Study
Source: PLoS One. 2016 Dec 15;11(12):e0168480. doi: 10.1371/journal.pone.0168480 (PMC5158313; doi:10.1371/journal.pone.0168480)
Supplement: S1 File — (DOCX) [file pone.0168480.s001.docx]

**S1 File: Supplementary Text and Figures for**

**“Eosinophil count is a common factor for complex metabolic and pulmonary traits and diseases: The LifeLines Cohort Study” by Amini M, et al.**

**Content: pages:**

**Review on previous studies** 2

a. Table A 2

**Methods**

I. Ascertainment of Type 2 Diabetes (T2D) in LifeLines 4

1. Subjects with a positive self-reported on diabetes 4
   1. Figure A 6
2. Subjects with a negative self-reported on diabetes 5
   1. Figure B 7

II. Ascertainment of asthma 8

- 1. Figure C 9

**Results**

a. Table B 10

- 1. Table C 11
  2. Table D 12
  3. Table E 13

**References** 14

**Review on previous studies**

| **Table A.** Previous studies on associations of eosinophil count with complex diseases and intermediate traits. | | | | | | |
| --- | --- | --- | --- | --- | --- | --- |
| **Class** | **Outcome** | **Author (year)** | **Population** | **Sample size** | **Type of study** | **Main result** |
| **Metabolic Syndrome** | **Obesity** | Meng W. *et al.*(2012) [1] | Chinese | 6,513 | Longitudinal cohort study | The eosinophil level were strong risk factor for obesity. |
|  |  | Kim D.J. *et al.*(2008) [2] | Korean | 15,654 | Cross-sectional | With elevation of the eosinophil count, the risk of obesity in Korean population was increased. |
|  | **Metabolic syndrome** | Babio N. *et al*. (2013) [3] | Spanish | 4,377 | Cross-sectional and Longitudianl studies (PREDIMED) | Participants in the upper quartile of eosinophil count had a greater prevalence of MetS than participants in the lower quartile. |
|  |  | [Meng W. *et al.* (2012)](http://www.ncbi.nlm.nih.gov/pubmed/?term=Association+between+Leukocyte+and+Metabolic+Syndrome+in+Urban+Han+Chinese%3A+A+Longitudinal+Cohort+Study) [1] | Chinese | 6,513 | Longitudinal cohort study | Eosinophil was not associated with MetS components. |
|  |  | Kim D. J. *et al.* (2008) [2] | Korean | 15,654 | Cross-sectional | Total leukocyte count and differential leukocyte count including eosinophil count were independently associated with MetS presence. |
|  |  | [Shim W. S. *et al.* (2006](http://www.ncbi.nlm.nih.gov/pubmed/16563549)) [4] | Korean | 822 | Cross-sectional | With increasing the number of MetS components, eosinophil count were increased in T2D patients. |
|  |  | Kim J. A. *et al.* (2006) [5] | Korean | 399 | Cross-sectional | No significant association between eosinophil count and MetS components. |
|  | **Type 2 Diabetes** | Zhu L. *et al.* (2013) [6] | Chinese | 9,111 | Cross-sectional | Eosinophil percentage was inversely associated with the risk of T2D and insulin resistance in normal glucose tolerance individuals. |
|  |  | Xu W. *et al.* (2013) [7] | Chinese | 150 | Case- control | Decreased eosinophil count reflect the presence of hyperglycemic emergency. |
|  |  | Meng W. *et al.* (2012) [1] | Chinese | 6,513 | Longitudinal cohort study | Eosinophil was not associated with hyperglycemia as a MetS component. |
|  |  | Gkrania-Klotsas E. *et al.* (2010) [8] | Caucasian | 8,647 T2D and 85,040 non-case | Systematic review | Eosinophil count were not significantly associated with risk of T2D; while, total leukocyte count, neutrophil and lymphocyte were associated. |
|  |  | Fukui M. *et al.* (2009) [9] | Japanese | 783 patients | Cross-sectional | Eosinophil count were positively associated with TG, albumin excretion rate in type 2 diabetic men; while, they were unrelated to albumin excretion in diabetic women. |
|  |  | Shim W. S. *et al.* (2006) [4] | Korean | 822 | Cross-sectional | With increasing the number of MetS components, eosinophil count were increased in type 2 diabetic patients. |
|  |  | Chung F. M. *et al.* (2005) [10] | Taiwanese | 1,480 | Cross-sectional | With progression in the diabetes nephropathy, the mean of eosinophil count increased. |
|  |  | Vozarova B. *et al.* (2002) [11] | Indian | 352 | Longitudinal study | No part of differential white blood cells including eosinophil count was predictive of diabetes either before or after adjustment for age, sex, and body fat. |
| **Cardiac Disease** | **Hypertension** | Meng W. *et al.* (2012) [1] | Chinese | 6,513 | Longitudinal cohort study | Eosinophil was not associated with hypertension as MetS component. |
|  |  | Kim D. J. *et al.* (2008) [2] | Korean | 15,654 | Cross-sectional | As eosinophil level was elevated the frequency of hypertension was also increased. |
|  | **Myocardial infarction** | Gudbjartsson D. F. *et al.* (2009) [12] | European | 6,650 cases and 40,621 control | Meta-GWAS | Eosinophil associated SNP rs3184504 also associated with an increased risk of myocardial infarction in six different populations. |
| **Pulmonary Disease** | **COPD** | Bafadhel M. *et al.* (2011) [13] | British | 145 | Longitudinal cohort study | There were similar increases in sputum and blood eosinophil numbers during COPD exacerbation. |
|  |  | Gorska K. *et al.* (2008) [14] | Polish | 17 | Observational | Higher eosinophil count were in induced sputum in COPD patients. |
|  |  | Siva R. *et al.* (2007) [15] | British | 82 | Randomized control trial | Modulation of eosinophilic airway inflammation have effect on outcome in COPD patients. |
|  |  | Hospers J. J. *et al.* (2000) [16] | Netherlands | 5,383 | Longitudinal cohort study | eosinophilia is associated with increased all-cause mortality in a study on asthma and COPD in a general population sample. |
|  |  | Mensinga T. T. *et al.* (1992) [17] | Netherlands | 4,242 | Longitudinal cohort study | An association of eosinophilia with lower levels of FEV1 is independent of the effect of cigarette smoking. |
|  | **Asthma** | Talina D. *et al.* (2015) [18] | Italian | 39 | Observational | Sputum eosinophilia were significantly associated with a greater decline in FEV_1_ in occupational asthma patients. |
|  |  | Hastie A. T. *et al.* (2013) [19] | American | 328 | Cross-sectional | Sputum eosinophil was significantly correlated with asthma severity. |
|  |  | Broekema M. *et al.* (2010) [20] | Netherlands | 47 | Cross-sectional | There was association between high eosinophil level in bronchial biopsies and accelerated FEV_1_ decline in adult patients with asthma. |
|  |  | Gudbjartsson D. F*. et al.* (2009) [12] | European | 7,996 cases and 44,890 control | Meta-GWAS | Eosinophil associated SNP rs1420101 also associated with asthma in ten different population. |
|  |  | Hospers J. J. *et al.* (2000) [16] | Netherlands | 5,383 | Longitudinal cohort study | eosinophilia is associated with increased all-cause mortality in a study on asthma and COPD in a general population sample. |
|  |  | Jatakanon A. *et al.* (2000) [21] | British | 15 | Case-control | There was significant correlation between increases in sputum eosinophils with asthma subsequent exacerbations and decreases in airway function including FEV_1_. |

**Methods**

**I. Ascertainment of T2D in LifeLines**

The approach for ascertainment of T2D in LifeLines is presented in Figures A and B. In the LifeLines cohort all participants filled in a structured questionnaire and were subjected to a medical examination at baseline. Four sources of ascertaining T2D were recorded in LifeLines: a) self-reported T2D, b) fasting glucose (FG), c) hemoglobin A1c (HbA1c), and d) anti-T2D medication use. The data were interpreted using two main categories of (A) subjects with a positive self-report of T2D, and (B) subjects who did not report a clinical history of T2D, but have a positive finding in favor of the diagnosis of T2D.

**a)** At baseline, all individuals who agreed to participate received a self-administered general questionnaire containing questions on demographic characteristics, presence of chronic diseases, and risk factors for chronic diseases. The questionnaire contained four questions on diabetes. Participants were asked if they had been diagnosed with diabetes previously (level 1), and if yes, additional questions on the type of diabetes (level 2) (type 1 diabetes, T2D, other types, I do not know), the year of diagnosis and type of treatment were asked (level 3) (no treatment, oral glucose-lowering medication, insulin injection, oral glucose-lowering medication and insulin injection together, and diet).

**b, c)** *FG and HbA1c:* FG and HbA1c (Roche, Germany) were measured using standard assays and procedures by LifeLines research and UMCG medical laboratories. Based on the latest guidelines on screening and diagnosis of T2D the threshold for detection of T2D was ≥7.0 mmol/L for FG and ≥6.5% for HbA1c [22-24].

**d)** *Medication use:* The questionnaire contained questions concerning anti- T2D medications use. ATC codes of anti-T2D medications used by participants (i.e. oral glucose-lowering medication or insulin) have been recorded by pharmacist students. Recorded ATC codes for anti-T2D medications in database were A10AB01-A10AB04-A10AB05-A10AB06-A10AC01-A10AC04-A10AD05-A10AD01-A10AE04-A10AE05-A10BA01-A10BA02-A10BB01-A10BB02-A10BB03-A10BB09-A10BB12-A10BD02-A10BG03-A10BH01.

**Elucidation**: Based on previous studies [25-29] combining self-reported data with alternative ascertainment source might contribute to a higher identification of T2D cases. In this study, T2D was defined as either self-reported T2D (Figure A), or in undiagnosed T2D it was based on FG≥7.0 mmol/L or HbA1c≥6.5% or recorded anti-T2D medication use (Figure B).

**I. A. Subjects with a positive self-reported on diabetes (Figure A):**

Self-reported diabetes resulted in six different scenarios when cross checked with medical records and FG and HbA1c measurements. These included:

*Scenario 1* (N=19): Individuals who reported they had diabetes and the type of diabetes was T2D but this was not supported based on self-reported type of treatment (missing). So, T2D in these individuals was diagnosed according to the recorded anti- T2D medication use or FG≥7.0 or HbA1c≥6.5, when data available.

*Scenario 2* (N=10): Individuals who reported they had diabetes and the type of diabetes was T2D but they were not under treatment. So, T2D was considered when participants reported use of anti- T2D medication or when participant had a FG≥7.0 or HbA1c≥6.5.

*Scenario 3* (N=252): Individuals who reported they had diabetes and the type of diabetes was T2D. Moreover, they reported to be on treatment with insulin, or tablet, or insulin and tablet together, or diet. They were considered as T2D patients.

*Scenario 4* (N=1): Individuals who reported they had diabetes but they did not know which type of diabetes. These individuals had not self-reported on type of treatment. So, T2D was considered based on the recorded anti- T2D medication use or FG≥7.0 or HbA1c≥6.5.

*Scenario 5* (N=0): Individuals who reported they had diabetes but did not know which type of diabetes. These individuals reported not to be on treatment. So, T2D was considered when participants used anti- T2D ATC codes medication or when participants had FG≥7.0 or HbA1c≥6.5.

*Scenario 6* (N=6): Individuals who reported they had diabetes but they did know which type of diabetes. Moreover, these individuals reported they used anti-hyperglycemic tablets, insulin, or tablets and insulin together, or diet; then, T2D was considered according to the anti- T2D ATC codes medication use or FG≥7.0 or HbA1c≥6.5.

Individuals who reported to have type 1 diabetes or other type of diabetes, they were controlled for the use of anti-T2D medication or FG≥7.0 or HbA1c≥6.5 and age of onset (>30 years old) to make sure they did not have T2D.

**I. B. Subjects with a negative self-reported on diabetes (Figure B*):***

There were 12,943 subjects who did not report having been diagnosed of T2D. We followed three different scenarios to confirm absence of T2D in these individuals:

*Scenario 1* (N=0): Individuals who reported they did not have diabetes, but they self-reported positive on type of diabetes and treatment for diabetes. These individuals were further checked if they reported use of anti-glycemic medication or if they had FG≥7.0 or HbA1c≥6.5. When all these were negative, we considered subjects as no T2D patients.

*Scenario 2* (N=213): Individuals who reported they did not have diabetes and they did not report on type of diabetes or treatments. We checked if they used anti- T2D medication or if they had FG≥7.0 or HbA1c≥6.5.

*Scenario 3* (N=6): Individuals who had missing answers on having diabetes or not. We considered T2D when participant was user of anti- T2D medication or participant had FG≥7.0 or HbA1c≥6.5.

**Figure A:** Ascertainment of self-reported T2D in the LifeLines cohort study.

**Database**

**N=13,301**

**Scenario 6**

**T2D**

**N=6**

**N=4**

Age>30

**N=2**

Age>30

**Tab N=0**

**Ins N=5**

**Tab&Ins N=1**

**Diet N=1**

**No Treat**

**N=2**

**Missing**

**N=2**

**Missing**

**N=2**

**Tab N=2**

**Ins N=1**

**Tab&Ins N=2**

**Diet N=3**

**Check for Med or FG≥7**

**Or HbA1c≥6.5**

**T1D**

**N=23**

**Scenario 4**

**T2D**

**N=1**

**N=1**

Age=33

**4) I do not know**

**N=11**

**3) Other type**

**N=27**

**1) T1D**

**N=24**

**53< age<7**

**Do you have diabetes?**

**(Level 1)**

**Diabetes=Yes/No**

**Yes: N=358**

**No: N= 12,865 (see Figure B)**

**Missing: 78 (see Figure B)**

**Which type of diabetes?**

**Options: 1-4**

**(level 2)**

**2) T2D**

**N=296**

**No Treat N= 0**

**Tab N=0**

**Ins N=20**

**Tab&Ins N=3**

**Diet N=0**

**No Treat**

**N=20**

**Tab N=156**

**Ins N=24**

**Tab&Ins N=31**

**Diet N=41**

**No Treat**

**N=17**

**Missing**

**N= 24**

**Which type of treatment?**

**(level 3)**

**Check for Med* or FG≥7 or HbA1c≥6.5**

**Check for Med or FG≥7 or HbA1c≥6.5**

**Check for Med or FG≥7 or HbA1c≥6.5**

**Scenario 3**

**T2D**

**N=252**

**Scenario 1**

**T2D**

**N=19**

**Scenario 2**

**T2D**

**N=10**

**Conclusion on diagnosis of diabetes**

**Scenario 5**

**T2D**

**N= 0**

**Check for Med or FG≥7 or HbA1c≥6.5**

**Med**

**N=22**

**FG≥7 or HbA1c≥6.5**

**N=22**

: means excluded as type 2 diabetes.

*Med: Recorded ATC codes medication use


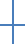


**Figure B:** Ascertainment of non-self-reported T2D in the LifeLines cohort study.

**T2D= 507/13,301=3.81%**

**ATC recorded medication= 264**

**Self -reported medication= 248**

**FG≥7= 291**

**HbA1c ≥ 6.5= 365**

**Diet= 49**

**ATC Codes T2D= A10AB01-A10AB04-A10AB05-A10AB06-A10AC01- A10AC04-A10AD05-A10AD01-A10AE04-A10AE05**

**A10BA01- A10BA02-A10BB01- A10BB02-A10BB03-A10BB09-A10BB12-A10BD02-A10BG03-A10BH01**

**Check for FG≥7 or HbA1c≥6.5**

**N= 213**

**5) Rest**

**N=12,846**

**Check for Med or FG≥7 or HbA1c≥6.5**

**Check for Med* or FG≥7 or HbA1c≥6.5**

**Missing**

**N=78**

**Check for MED**

**N= 0**

**2) T2D**

**N=1**

**1) T1D**

**N=0**

**Diabetes=No**

**N=12,865**

**Database**

**N=13,301**

**Do you have diabetes?**

**(Level 1)**

**Which type of diabetes?**

**Options: 1-5**

**(level 2)**

**4) I do not know**

**N=8**

**3) Other type N=10**

**No Treat N=0**

**Tablet N=0**

**Insulin N=0**

**Tab&Ins N=0**

**Diet N=0**

**Which type of treatment?**

**(level 3)**

**Scenario 3**

**T2D**

**N=6**

**Scenario 2**

**T2D**

**N= 213**

**Scenario 1**

**T2D**

**N=0**

**Conclusion on diagnosis of diabetes**


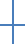


*Med: Recorded ATC codes medication use

**II. Ascertainment of asthma (Figure C):**

In brief, the definition of asthma in the cohort was based on a self-reported clinical diagnosis of asthma or two and more symptoms of asthma and asthma medication use. Figure C shows ascertainment of asthma in the LifeLines cohort study. Ascertainment of asthma was based on three available sources of a) self-reported asthma, b) symptoms of asthma, and c) recorded ATC codes medication use in database.

***a)*** *Self-reported asthma:* We defined self-reported asthma as positive answers to both questions “Have you ever had asthma?” and “If so, this was confirmed by a doctor?’’.

***b)*** *Asthma* *symptoms:* Individuals were considered to have asthma symptoms if two or three of the following questions were answered positively: “Have you ever suffered from wheezing?”, “Have you ever had an attack of shortness of breath at rest?”, “Have you ever been woken by an attack of shortness of breath?”.

***c)*** *Asthma medications:* Asthma medications included any of the following medications categories: beta-2-adrenoreceptor agonists, combimed (adrenergics in combination with corticosteroids or other drugs, excluding anticholinergics), inhaled corticosteroids, anticholinergics, theofyllines, montelukast, cromoglycates. Asthma medication and related ATC codes in database were recorded by pharmacist students and they were as following: R03AC02, R03AC03, R03AC12, R03AC13, R03CC02, R03CC03, R03AK03, R03AK06, R03AK07, R03BAC01, R03BA02, R03BA03, R03BA04, R03BA05, R03BA08, R03BB01, R03BB02, R03BB04, R03BC01, R03BC03, R03DA04, R03DC003.

**Figure C:** Asthma disease ascertainment in the LifeLines cohort study.

**Asthma medication**

**Asthma symptoms**

**No asthma**

**N=1,741**

**Asthma**

**N= 47**

**Asthma= 967/13,301= 7.27%**

**Asthma**

**N= 946**

**Check for asthma Medication use**

**Wheeze &**

**attack at rest &**

**woken by an attack**

**N=292**

**Wheeze**

**&**

**attack at rest**

**N=590**

**Wheeze**

**&**

**woken by an attack**

**N=404**

**Attack at rest**

**&**

**Woken by an attack**

**N=462**

**Database**

**N=13,301**

**Asthma self-reported**

**Self-reported of asthma**

**&**

**Doctor diagnosis of asthma**

**N= 946**

**Diagnosed Asthma**

**ATC medication=** R03AC02, R03AC03, R03AC12, R03AC13, R03CC02, R03CC03, R03AK03, R03AK06, R03AK07, R03BAC01, R03BA02, R03BA03, R)#BA04, R03BA05, R03BA08, R03BB01, R03BB02, R03BB04, R03BC01, R03BC03, R03DA04, R03DC003

**Results**

| **Table B.** Multivariate regression results of basic model and full models of ln-transformed eosinophil count with studied intermediate traits and diseases in metabolic class. | | | | | | | |
| --- | --- | --- | --- | --- | --- | --- | --- |
|  | | **Model I** | **Model II** | | **Model III** | | |
|  | | **Sβ ± SE** | | | | | |
| ln-transformed BMI | | 0.04 ± 0.002^***^ | | 0.04 ± 0.002^***^ | | | 0.005 ± 0.002 |
| ln-transformed Triglycerides | | 0.07 ± 0.007^***^ | | 0.06 ± 0.007^***^ | | | 0.009 ± 0.007 |
| Total Cholesterol | | 0.04 ± 0.003^***^ | | 0.04 ± 0.003^***^ | | | 0.04 ± 0.003^***^ |
| High-Density Lipoprotein | | -0.06 ± 0.004^***^ | | -0.05 ± 0.004^***^ | | | -0.005 ± 0.004 |
| Low-Density Lipoprotein | | 0.05 ± 0.004^***^ | | 0.04 ± 0.004^***^ | | | 0.04 ±0.004^***^ |
| HbA1c | | 0.05 ± 0.006^***^ | | 0.04 ± 0.006^***^ | | | 0.01 ±0.007 |
| Fasting glucose | | 0.02 ± 0.013 | | 0.008 ± 0.013 | | | 0.01 ± 0.013 |
|  | | **OR (95%CI)** | | | | | |
| Obesity | 1.18 (1.09-1.28)^***^ | | 1.18 (1.09-1.28) ^***^ | | | 1.01 (0.92-1.09) | |
| Metabolic Syndrome | 1.29 (1.19-1.39)^***^ | | 1.29 (1.19-1.39) ^***^ | | | 1.07 (0.98-1.162) | |
| Type 2 diabetes | 1.12 (0.98-1.28) | | 1.05 (0.91-1.20) | | | 0.93 (0.80-1.08) | |
| **^***^** Associations are significant at a p-value<0.00076  **Sβ:** standardized coefficient**, BMI**: body mass index, **HbA1c:** hemoglobin A1c.  **#1:** **Model I** All analysis on intermediate traits and diseases were adjusted for confounding effect of age, age^2^, and sex.  **Model II** All analysis on intermediate traits were adjusted for confounding effect of age, age^2^, sex, BMI (with exception on BMI) and smoking habit. Analysis models on diseases were adjusted for confounding effect of age, age^2^, sex, and smoking habit; as well as, BMI also was adjusted on type 2 diabetes. **Model III** All analysis on intermediate traits were adjusted for confounding effect of age, age^2^, sex, BMI (with exception on BMI), smoking habit, and leukocytes count. Analysis models on diseases were adjusted for confounding effect of age, age^2^, sex, smoking habit, and leukocytes count; as well as, BMI was adjusted on type 2 diabetes.  **#2:** Standardized coefficient (Sβ) means a one-standard deviation higher eosinophil count would result in a βxSD change in the outcome variables. | | | | | | | |

| **Table C.** Multivariate regression results of basic model and full models of ln-transformed eosinophil count with studied intermediate traits and diseases in cardiac class. | | | |
| --- | --- | --- | --- |
|  | **Model I** | **Model II** | **Model III** |
|  | **Sβ ± SE** | | |
| ln-transformed SBP | 0.02 ± 0.002 | 0.007 ± 0.002 | -0.02 ± 0.002 |
| ln-transformed DBP | 0.02 ± 0.002 | 0.01 ± 0.002 | -0.005 ± 0.002 |
| ln-transformed MAP | 0.02 ± 0.002 | 0.009 ± 0.002 | -0.01 ± 0.002 |
| Pulse Pressure | 0.01 ± 0.167 | 0.003 ± 0.168 | -0.02 ± 0.174 |
| eGFR | -0.002 ± 0.167 | -0.003 ± 0.167 | -0.002 ± 0.172 |
| ln-transformed UACR | -0.02 ± 0.012 | -0.01 ± 0.016 | -0.02 ± 0.015 |
|  | **OR (95%CI)** | | |
| Hypertension | 1.06 (0.99-1.14) | 1.02 (0.95-1.10) | 0.95 (0.88-1.02) |
| Myocardial Infarction | 1.50 (1.16-1.94) | 1.46 (1.12-1.90) | 1.27 (0.96-1.69) |
| **^***^** Associations are significant at a p-value<0.00076.  **Sβ:** standardized coefficient**, SBP:** systolic blood pressure**, DBP:** diastolic blood pressure, **MAP:** mean arterial pressure, **eGFR:** estimated glomerular filtration rate, **UACR:** Urine Albumin-to-Creatinine Ratio.  **#1:** **Model I** All analysis on intermediate traits and diseases were adjusted for confounding effect of age, age^2^, and sex. **Model II** All analysis on intermediate traits and diseases were adjusted for confounding effect of age, age^2^, sex, BMI and smoking habit. Additionally, SBP, DBP, height, overweight and obesity were adjusted in eGFR and UACR models. **Model III** All analysis on intermediate traits and diseases were adjusted for confounding effect of age, age^2^, sex, BMI, smoking habit and leukocytes count. Additionally, SBP, DBP, height, overweight and obesity were adjusted in eGFR and UACR models.  **#2:** Standardized coefficient (Sβ) means a one-standard deviation higher eosinophil count would result in a β×SD change in the outcome variables. | | | |

| **Table D.** Multivariate regression result of basic model and full models of ln-transformed eosinophil count with studied intermediate traits and diseases in pulmonary class. | | | |
| --- | --- | --- | --- |
|  | **Model I** | **Model II** | **Model III** |
|  | **Sβ ± SE** | | |
| FEV_1_ | -0.05 ± 0.008^***^ | -0.05 ± 0.007^***^ | -0.04 ± 0.007^***^ |
| FEV_1_/FVC | -0.09 ± 0.001^***^ | -0.09 ± 0.001^***^ | -0.08 ± 0.001^***^ |
|  | **OR (95%CI)** | | |
| COPD | 1.36 (1.22-1.50)^***^ | 1.40 (1.25-1.56) ^***^ | 1.28 (1.14-1.43)^***^ |
| Asthma | 1.85 (1.65-2.07)^***^ | 1.81 (1.61-2.03) ^***^ | 1.80 (1.60-2.03)^***^ |
| **^***^** Associations are significant at a p-value<0.00076.  **Sβ:** standardized coefficient, **FEV_1_:** Forced expiratory volume in one second, **FVC:** Forced vital capacity.  **#1:** **Model I** All analysis on intermediate traits and diseases were adjusted for confounding effect of age, age^2^, and sex. **Model II** All analysis models on intermediate traits and diseases were adjusted for confounding effect of age, age^2^, sex, smoking habit, and height. **Model III** All analysis models on intermediate traits and diseases were adjusted for confounding effect of age, age^2^, sex, smoking habit, height, and leukocytes count.  **#2**: Standardized coefficient (Sβ) means a one-standard deviation higher eosinophil count would result in a β×SD change in the outcome variables. | | | |

| **Table E.** Partial correlation results of ln-transformed eosinophil count with the studied intermediate traits in metabolic, cardiac and pulmonary classes of overall population.^††^ | | | |
| --- | --- | --- | --- |
| **Classes** | **Intermediate Traits** | **Partial Correlation r^#^** | **p-value** |
| Metabolic | ln-transformed BMI | 0.04 | 8.0×10^-6^ |
|  | ln-transformed Triglycerides | 0.07 | 3.2×10^-12^ |
|  | Total Cholesterol | 0.04 | 7.0×10^-6^ |
|  | High-Density Lipoprotein | -0.06 | 1.2×10^-8^ |
|  | Low-Density Lipoprotein | 0.05 | 6.3×10^-7^ |
|  | HbA1c | 0.04 | 6.0×10^-6^ |
|  | Fasting glucose | 0.004 | 0.726 |
| Cardiac | ln-transformed SBP | 0.009 | 0.345 |
|  | ln-transformed DBP | 0.01 | 0.244 |
|  | ln-transformed MAP | 0.01 | 0.250 |
|  | Pulse Pressure | 0.003 | 0.749 |
|  | eGFR | -0.003 | 0.709 |
|  | ln-transformed UACR | -0.01 | 0.252 |
| Pulmonary | FEV_1_ | -0.08 | 6.9×10^-18^ |
|  | FEV_1_/FVC | -0.10 | 1.2×10^-27^ |
| **BMI**: body mass index, **HbA1c:** hemoglobin A1c, **SBP:** systolic blood pressure**, DBP:** diastolic blood pressure, **MAP:** mean arterial pressure, **eGFR:** estimated glomerular filtration rate, **UACR:** Urine Albumin-to-Creatinine Ratio, **FEV_1_:** Forced expiratory volume in one second, **FVC:** Forced vital capacity.  **^#^**All analysis on intermediate traits were adjusted for confounding effect of age, age^2^, sex, BMI (with exception on BMI) and smoking habit. Additionally, SBP, DBP, height, overweight and obesity were adjusted in eGFR and UACR models. | | | |

^††^ In comply to reviewer’s suggestion, we present partial correlation analyses to evaluate the relationship between eosinophil count and the studied intermediate outcomes while controlling for covariates effect. Similar to the results obtained from multivariate regression analyses, there was a significant association between eosinophil count and intermediate traits of metabolic and pulmonary classes. A higher eosinophil count was correlated to a higher levels of BMI, TG, TC, LDL, HbA1c and a lower levels of HDL, FEV1, and FEV1/FVC. Controlling for the effect of age, age^2^, sex, BMI, smoking habit, height, SBP, DBP, overweight and obesity had ignorable influence on the strength of the relation between variables.

**References**

1. Meng W, Zhang C, Zhang Q, Song X, Lin H, Zhang D, et al. Association between leukocyte and metabolic syndrome in urban Han Chinese: a longitudinal cohort study. PLoS One 2012, 7(11):e49875.

2. Kim DJ, Noh JH, Lee BW, Choi YH, Chung JH, Min YK, et al. The associations of total and differential white blood cell counts with obesity, hypertension, dyslipidemia and glucose intolerance in a Korean population. J Korean Med Sci 2008, 23(2):193-198.

3. Babio N, Ibarrola-Jurado N, Bullo M, Martinez-Gonzalez M, Warnberg J, Salaverria I, et al. White blood cell counts as risk markers of developing metabolic syndrome and its components in the PREDIMED study. PLoS One 2013, 8(3):e58354.

4. Shim WS, Kim HJ, Kang ES, Ahn CW, Lim SK, Lee HC, et al. The association of total and differential white blood cell count with metabolic syndrome in type 2 diabetic patients. Diabetes Res Clin Pract 2006, 73(3):284-291.

5. Kim JA, Choi YS, Hong JI, Kim SH, Jung HH, Kim SM. Association of metabolic syndrome with white blood cell subtype and red blood cells. Endocr J 2006, 53(1):133-139.

6. Zhu L, Su T, Xu M, Xu Y, Li M, Wang T, et al. Eosinophil inversely associates with type 2 diabetes and insulin resistance in Chinese adults. PLoS One 2013, 8(7):e67613.

7. Xu W, Wu HF, Ma SG, Bai F, Hu W, Jin Y, et al. Correlation between peripheral white blood cell counts and hyperglycemic emergencies. Int J Med Sci 2013, 10(6):758-765.

8. Gkrania-Klotsas E, Ye Z, Cooper AJ, Sharp SJ, Luben R, Biggs ML, et al. Differential white blood cell count and type 2 diabetes: systematic review and meta-analysis of cross-sectional and prospective studies. PLoS One 2010, 5(10):e13405.

9. Fukui M, Tanaka M, Hamaguchi M, Senmaru T, Sakabe K, Shiraishi E, et al. Eosinophil count is positively correlated with albumin excretion rate in men with type 2 diabetes. Clin J Am Soc Nephrol 2009, 4(11):1761-1765.

10. Chung FM, Tsai JC, Chang DM, Shin SJ, Lee YJ. Peripheral total and differential leukocyte count in diabetic nephropathy: the relationship of plasma leptin to leukocytosis. Diabetes Care 2005, 28(7):1710-1717.

11. Vozarova B, Weyer C, Lindsay RS, Pratley RE, Bogardus C, Tataranni PA. High white blood cell count is associated with a worsening of insulin sensitivity and predicts the development of type 2 diabetes. Diabetes 2002, 51(2):455-461.

12. Gudbjartsson DF, Bjornsdottir US, Halapi E, Helgadottir A, Sulem P, Jonsdottir GM, et al. Sequence variants affecting eosinophil numbers associate with asthma and myocardial infarction. Nat Genet 2009, 41(3):342-347.

13. Bafadhel M, McKenna S, Terry S, Mistry V, Reid C, Haldar P, et al. Acute exacerbations of chronic obstructive pulmonary disease: identification of biologic clusters and their biomarkers. Am J Respir Crit Care Med 2011, 184(6):662-671.

14. Gorska K, Krenke R, Korczynski P, Kosciuch J, Domagala-Kulawik J, Chazan R. Eosinophilic airway inflammation in chronic obstructive pulmonary disease and asthma. 2008, 59(Suppl 6):261-270.

15. Siva R, Green RH, Brightling CE, Shelley M, Hargadon B, McKenna S, et al. Eosinophilic airway inflammation and exacerbations of COPD: a randomised controlled trial. Eur Respir J 2007, 29(5):906-913.

16. Hospers JJ, Schouten JP, Weiss ST, Postma DS, Rijcken B. Eosinophilia is associated with increased all-cause mortality after a follow-up of 30 years in a general population sample. Epidemiology 2000, 11(3):261-268.

17. Mensinga TT, Schouten JP, Weiss ST, Van der Lende R. Relationship of skin test reactivity and eosinophilia to level of pulmonary function in a community-based population study. Am Rev Respir Dis 1992, 146(3):638-643.

18. Talini D, Novelli F, Bacci E, Bartoli M, Cianchetti S, Costa F, et al. Sputum eosinophilia is a determinant of FEV1 decline in occupational asthma: results of an observational study. BMJ Open 2015, 5(1):e005748-2014-005748.

19. Hastie AT, Moore WC, Li H, Rector BM, Ortega VE, Pascual RM, et al. Biomarker surrogates do not accurately predict sputum eosinophil and neutrophil percentages in asthmatic subjects. J Allergy Clin Immunol 2013, 132(1):72-80.

20. Broekema M, Volbeda F, Timens W, Dijkstra A, Lee NA, Lee JJ, et al. Airway eosinophilia in remission and progression of asthma: accumulation with a fast decline of FEV(1). Respir Med 2010, 104(9):1254-1262.

21. Jatakanon A, Lim S, Barnes PJ. Changes in sputum eosinophils predict loss of asthma control. Am J Respir Crit Care Med 2000, 161(1):64-72.

22. The International Expert Committee. International expert committee report on the role of the A1c assay in the diagnosis of diabetes. Diabetes Care 2009, 32.

23. Saudek CD, Herman WH, Sacks DB, Bergenstal RM, Edelman D, Davidson MB. A new look at screening and diagnosing diabetes mellitus. J Clin Endocrinol Metab 2008, 93(7):2447-2453.

24. American Diabetes Association: Standards of medical care in diabetes--2012. Diabetes Care 2012, 35 Suppl 1:S11-63.

25. Ngo DL, Marshall LM, Howard RN, Woodward JA, Southwick K, Hedberg K. Agreement between self-reported information and medical claims data on diagnosed diabetes in Oregon's Medicaid population. J Public Health Manag Pract 2003, 9:542-545.

26. Okura Y, Urban LH, Mahoney DW, Jacobsen SJ, Rodeheffer RJ. Agreement between self-report questionnaires and medical record data was substantial for diabetes, hypertension, myocardial infarction and stroke but not for heart failure. J Clin Epidemiol 2004, 57:1096-1103.

27. Robinson JR, Young TK, Roos LL, Gelskey DE. Estimating the burden of disease. Comparing administrative data and self-reports. Med Care 1997, 35:932-947.

28. Sluijs I, van der ADL, Beulens JWJ, Spijkerman AMW, Ros MM, Grobbee DE, et al. Ascertainment and verification of diabetes in the EPIC-NL study. Neth J Med 2010, 68(7):333-339.

29. Skinner KM, Miller DR, Lincoln E, Lee A, Kazis LE. Concordance between respondent self-reports and medical records for chronic conditions: experience from the Veterans Health Study. J Ambul Care Manage 2005, 28(2):102-110.
